# CHD4 regulates the DNA damage response and RAD51 expression in glioblastoma

**DOI:** 10.1038/s41598-019-40327-w

**Published:** 2019-03-14

**Authors:** Lisa D. McKenzie, John W. LeClair, Kayla N. Miller, Averey D. Strong, Hilda L. Chan, Edward L. Oates, Keith L. Ligon, Cameron W. Brennan, Milan G. Chheda

**Affiliations:** 1 Division of Oncology, Department of Medicine, Washington University School of Medicine, St. Louis, MO USA; 2 Department of Pathology, Brigham and Women’s Hospital, Harvard Medical School, Boston Children’s Hospital, and Dana Farber Cancer Institute, Boston, MA USA; 3 Department of Neurosurgery, Memorial Sloan Kettering Cancer Center, New York, NY USA; 4 Department of Neurology, Washington University School of Medicine, St. Louis, MO USA

## Abstract

Glioblastoma (GBM) is a lethal brain tumour. Despite therapy with surgery, radiation, and alkylating chemotherapy, most people have recurrence within 6 months and die within 2 years. A major reason for recurrence is resistance to DNA damage. Here, we demonstrate that CHD4, an ATPase and member of the nucleosome remodelling and deactetylase (NuRD) complex, drives a component of this resistance. CHD4 is overexpressed in GBM specimens and cell lines. Based on The Cancer Genome Atlas and Rembrandt datasets, CHD4 expression is associated with poor prognosis in patients. While it has been known in other cancers that CHD4 goes to sites of DNA damage, we found CHD4 also regulates expression of RAD51, an essential component of the homologous recombination machinery, which repairs DNA damage. Correspondingly, CHD4 suppression results in defective DNA damage response in GBM cells. These findings demonstrate a mechanism by which CHD4 promotes GBM cell survival after DNA damaging treatments. Additionally, we found that CHD4 suppression, even in the absence of extrinsic treatment, cumulatively increases DNA damage. Lastly, we found that CHD4 is dispensable for normal human astrocyte survival. Since standard GBM treatments like radiation and temozolomide chemotherapy create DNA damage, these findings suggest an important resistance mechanism that has therapeutic implications.

## Introduction

Glioblastoma (GBM) is the most common and aggressive brain tumor^1^. Treatment is surgery, radiation and the alkylating chemotherapy, temozolomide. After treatment, tumour recurrence is almost inevitable and on average occurs within 6 months^2,3^. Most patients die within 2 years^4^. Here, we have focused on a potential way to improve DNA damaging therapies by targeting chromodomain helicase DNA binding protein 4 (CHD4).

CHD4 is a highly conserved protein that is the core ATPase subunit of the nucleosome remodelling and deacetylase (NuRD) complex^5^. NuRD transcriptionally represses and activates genes^6^, arrests cell cycle progression at the G1/S transition^7,8^, and facilitates lineage commitment during embryonic development^9,10^. The NuRD complex can either promote or suppress tumourigenesis, depending on the context^11^. However, we know less about the role of CHD4 in cancer. Recent studies suggest CHD4 has several potential oncogenic and resistance-driving activities in multiple cell types. For example, somatic mutations in the CHD4 gene occur in approximately 20% of serous endometrial cancers, over half of which are located in its ATPase domain^12^. Overexpression of CHD4 is also associated with poor prognosis in non small-cell lung cancer (NSCLC)^13^, hepatocellular carcinoma (HCC)^14^ and colorectal cancer^15^. In colorectal cancer, CHD4 promotes the recruitment of DNA methyltransferases to tumour suppressor gene promoters, thereby repressing their expression and promoting tumourigenesis^15^. We previously found CHD4 is required to maintain GBM tumour initiating cell morphology and stem cell marker expression^16^. Therefore, CHD4 can promote cancer in multiple cell types.

CHD4 plays important roles in genome integrity by regulating signalling and repair after DNA damage^11,17–20^. In response to ionizing radiation or oxidative stress, CHD4 and the NuRD complex are rapidly recruited to sites of DNA damage through CHD4 association with Poly(ADP-ribose) polymerase 1 (PARP1). There, CHD4 helps create a repressive chromatin structure to prevent transcription of damaged genes^15,18^. Outside of its interaction with NuRD members, CHD4 is also recruited to the sites of DNA damage by RING finger ubiquitin ligase 8 (RNF8), which promotes assembly of DNA repair factors such as RNF168 and BRCA1^19^. Lastly, in response to DNA damage, the DNA damage response (DDR) kinases ATM^21^ and ATR^22^ phosphorylate CHD4. In turn, CHD4 also phosphorylates ATM in response to DNA damage^23^. Thus, CHD4 may be required for DNA repair and cell survival through multiple mechanisms.

CHD4 expression also promotes resistance to chemotherapeutic agents in some cancers. CHD4 contributes to cisplatin resistance in BRCA2-mutant breast cancers, by acting in an homologous recombination (HR)-independent manner^24^. In addition, CHD4 depletion in acute myeloid leukaemia (AML) cell lines increases sensitivity to cytarabine and daunorubicin^23^. These treatment resistance mechanisms are related to the role of CHD4 in DNA damage repair. However, given the multifaceted roles of CHD4, it is also likely that whether or not it drives resistance, and how it does this, is highly context dependent. We set out to explore the relevance of CHD4 to DNA damage response in GBM since DNA damage with radiation and alkylating chemotherapy has been the backbone of GBM treatment for decades.

Here, we report that CHD4 is overexpressed in GBM patient samples and cell lines, and that high expression of CHD4 correlates with poorer survival. We also demonstrate that survival of GBM cells, but not normal human astrocytes, depends upon CHD4. We provide evidence that CHD4 depletion causes DNA damage in GBM cell lines, even in the absence of exogenous DNA damaging agents, and that this may be due to decreased expression of RAD51. Finally, we show that CHD4 directly binds to the RAD51 promoter, and loss of CHD4 results in decreased activity at this promoter. Together, these data suggest a new manner by which CHD4 promotes the DDR response: through its direct regulation of RAD51. As such, CHD4 overexpression in GBM may promote cell survival and resistance to radiation, the mainstay of GBM treatment.

## Results

### CHD4 is highly expressed in GBM and is associated with poor patient survival

**Figure 1:**
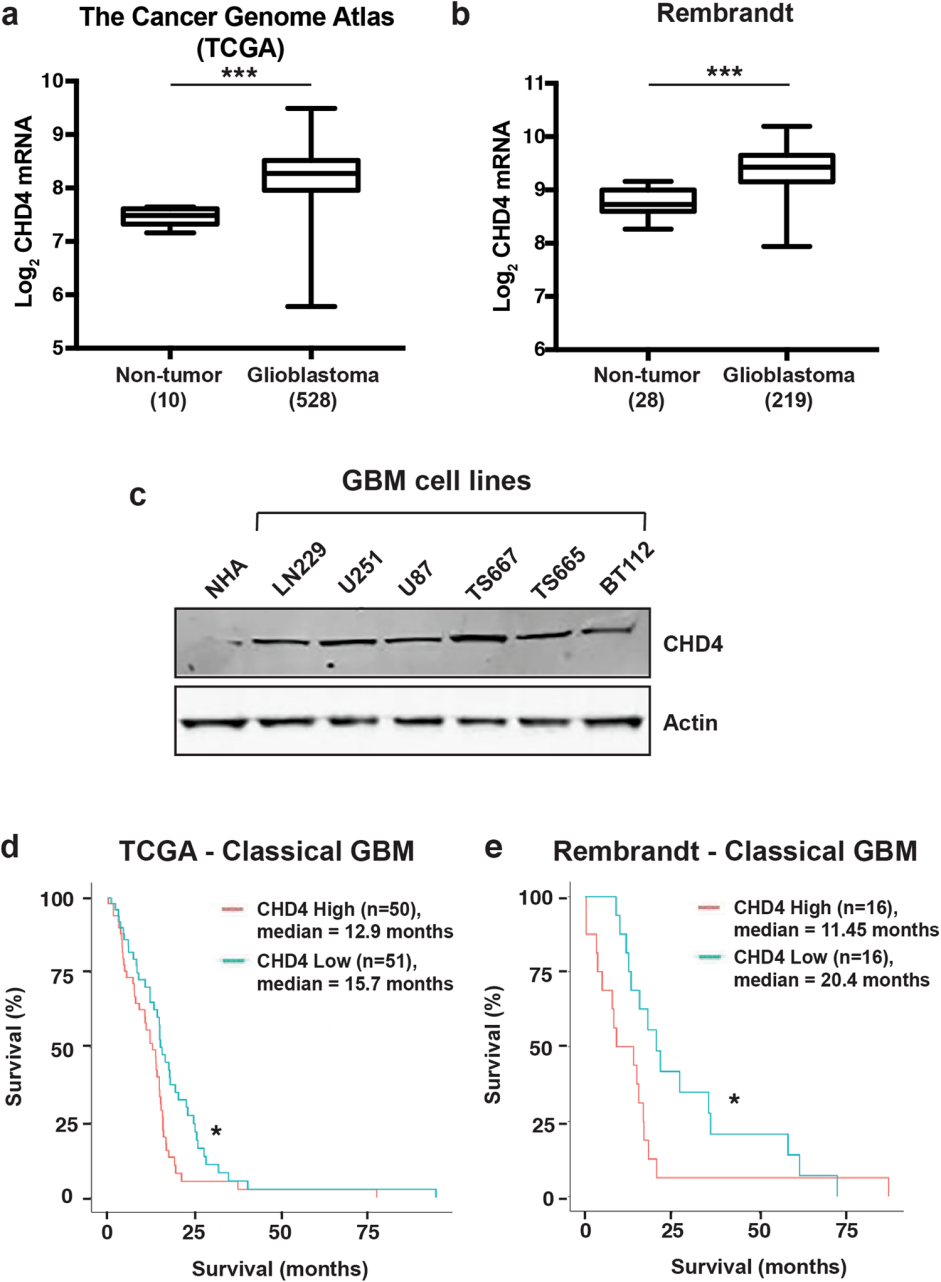
CHD4 is overexpressed in GBM and is associated with worse survival. (**a**) CHD4 mRNA expression in non-tumour (n = 10) and GBM patient samples (n = 526) in The Cancer Genome Atlas (TCGA). *p* = 2.2e-05. (**b**) CHD4 mRNA expression in non-tumour (n = 28) and GBM (n = 219) patient samples in the NCI Rembrandt repository. *p* = 2.3e-14. (**c**) Immunoblot for CHD4 in normal human astrocytes (NHA) and GBM cell lines. Full-length blots are presented in Supplementary Fig. 3. (**d**,**e**) Kaplan-Meier survival curves of GBM (classical subtype) patients with low CHD4 mRNA expression (CHD4 Low) and high CHD4 mRNA expression (CHD4 High) from TCGA (*p* = 0.0231) (**d**) and the Rembrandt (*p* = 0.0079) (**e**) data. Data are shown as the mean ± SEM (**a**,**b**). Significance was analysed with pairwise t-test with Bonferroni correction (**a**,**b**) or log-rank test (**d**,**e**). *p ≤ 0.05; **p ≤ 0.001; ***p ≤ 0.0001.

We analysed CHD4 mRNA expression data from The Cancer Genome Atlas (TCGA)^25^ and Rembrandt^26^ patient datasets using the GlioVis data portal^27^ to determine relative CHD4 expression in brain tumours. CHD4 mRNA is significantly upregulated in GBM patients (n = 528) compared with non-tumour controls (n = 10) in the TCGA dataset (*p* = 2.2e-05) (Fig. 1a). Similarly, CHD4 mRNA expression is significantly upregulated in GBM (n = 219, *p* = 2.3e-14) compared with non-tumour patient samples (n = 28) in the Rembrandt dataset (Fig. 1b). We compared CHD4 protein expression in multiple GBM cell lines (LN229, U251 and U87), tumour initiating cells (TS667, TS665 and BT112), and normal human astrocytes (NHA). Consistent with the patient data, CHD4 was more highly expressed in GBM cell lines compared with NHA (Fig. 1c). We then investigated whether high CHD4 expression correlates with poorer survival in patients by analysing patient survival data from the TCGA and Rembrandt datasets. We focused on the classical GBM subtype, because it is the most common type of GBM. Classical GBM patients in the TCGA dataset with CHD4 mRNA expression in the highest quartile (n = 50) have a significantly lower median survival (12.9 months) than patients with CHD4 mRNA expression in the lowest quartile (n = 51; 15.7 months), *p* = 0.0231 (Fig. 1d); these results did not change when we excluded patients with the glioma-CpG island methylator phenotype (G-CIMP) (n = 2). We found similar outcomes in the Rembrandt dataset, with classical GBM patients with high CHD4 expression (n = 16) having a significantly lower median survival (11.45 months) compared with classical subtype patients with low CHD4 mRNA expression (n = 16; 20.4 months), *p* = 0.0079 (Fig. 1e). Together, these findings demonstrate that CHD4 is highly expressed in GBM compared with normal brain and that, within classical GBM, CHD4 expression is associated with poor survival. CHD4 expression was significantly higher to a similar degree in all subclasses of GBM. However, the association of high CHD4 expression and poor survival was only observed in the classical GBM subtype.

### CHD4 is required for GBM cell viability

**Figure 2:**
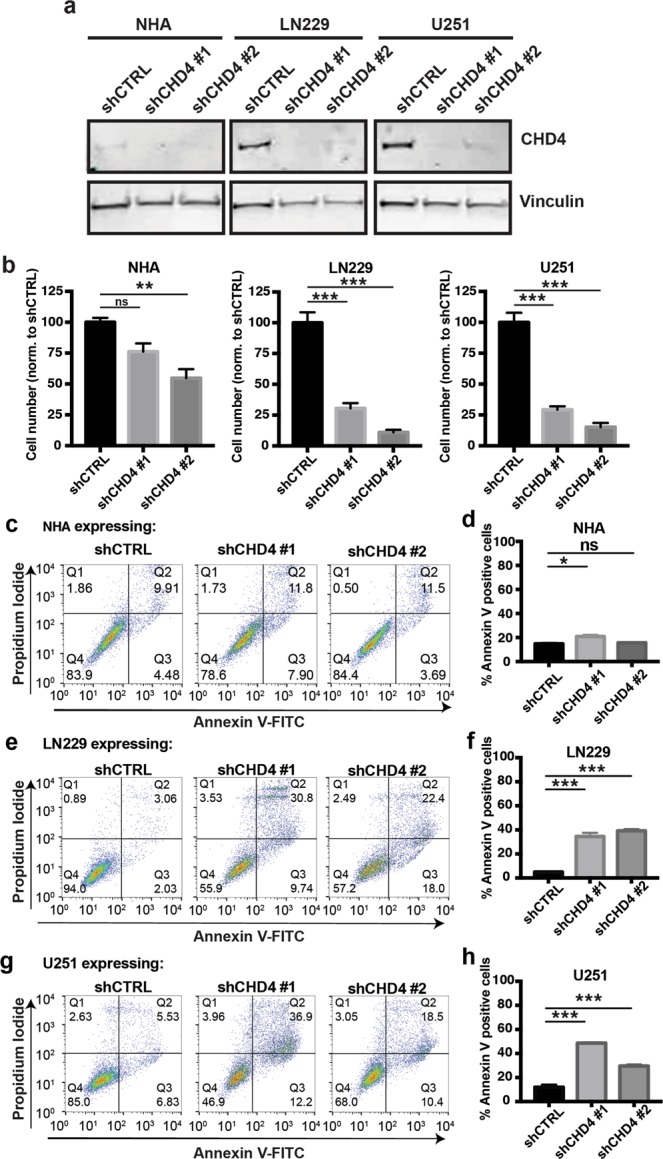
CHD4 suppression kills glioma cells but not normal astrocytes. (**a**) Immunoblot for CHD4 expression after suppression with indicated shRNAs in normal human astrocytes (NHA), and glioma cell lines, LN229 and U251. Full-length blots are presented in Supplementary Fig. 3. (**b**) Relative cell number after CHD4 suppression, normalized to non-targeting control, at 5 days post-shRNA transduction (NHA: shCTRL vs. shCHD4 #1, *p* = 0.0706, shCTRL vs. shCHD4 #2, *p* = 0.0042; LN229: shCTRL vs. shCHD4 #1, *p* = 0.0003, shCTRL vs. shCHD4 #2, *p* < 0.0001; U251: shCTRL vs. shCHD4 #1, *p* = 0.0002, shCTRL vs. shCHD4 #2, *p* = < 0.0001). (**c**) AnnexinV and propidium iodide (PI) labelling of NHAs 5 days after shRNA transduction. (**d**) Quantification of Annexin V positive cells from (**c**) (shCTRL vs. shCHD4 #1, *p* = 0.0129, shCTRL vs. shCHD4 #2, *p* = 0.076). (**e**) AnnexinV and PI labelling of LN229 cells 5 days after shRNA transduction (**f**) Quantification of Annexin V positive cells from (**e**) (shCTRL vs. shCHD4 #1, *p* < 0.0001, shCTRL vs. shCHD4 #2, *p* < 0.0001). (**g**) AnnexinV and PI labelling of U251 cells 5 days after shRNA transduction. (**h**) Quantification of Annexin V positive cells from (**g**) (shCTRL vs. shCHD4 #1, *p* < 0.0001, shCTRL vs. shCHD4 #2, *p* = 0.0003). Data are shown as mean ± SEM. ns = not significant; *p ≤ 0.05; **p ≤ 0.001; ***p ≤ 0.0001. All data is representative of three independent experiments (with three technical replicates each).

To test the effects of CHD4 on cell survival in GBM, we used multiple shRNAs targeting CHD4 in GBM cell lines (LN229 and U251) and NHAs. We focused on cell lines because tumour initiating cells differentiate after CHD4 suppression^16^, which confounds interpretation of proliferation and viability effects outside a general state change effect. All three cell lines transduced with shRNAs expressed less CHD4 protein when compared with the non-targeting control (shCTRL) (Fig. 2a). While CHD4 suppression mildly decreased NHA cell viability, it significantly decreased viability in LN229 and U251 cells (Fig. 2b). We then performed annexin V and propidium iodide (PI) staining followed by flow cytometry to determine whether this decreased cell number was due to increased apoptosis. We found that both shRNAs markedly increased the percentage of annexin V-positive LN229 and U251 cells (Fig. 2e–h). In contrast, CHD4 suppression had little effect on NHAs (Fig. 2c,d). These results demonstrate that GBM cell lines are more dependent on CHD4 for survival than normal cells.

### CHD4 is required for the GBM DNA damage response

**Figure 3:**
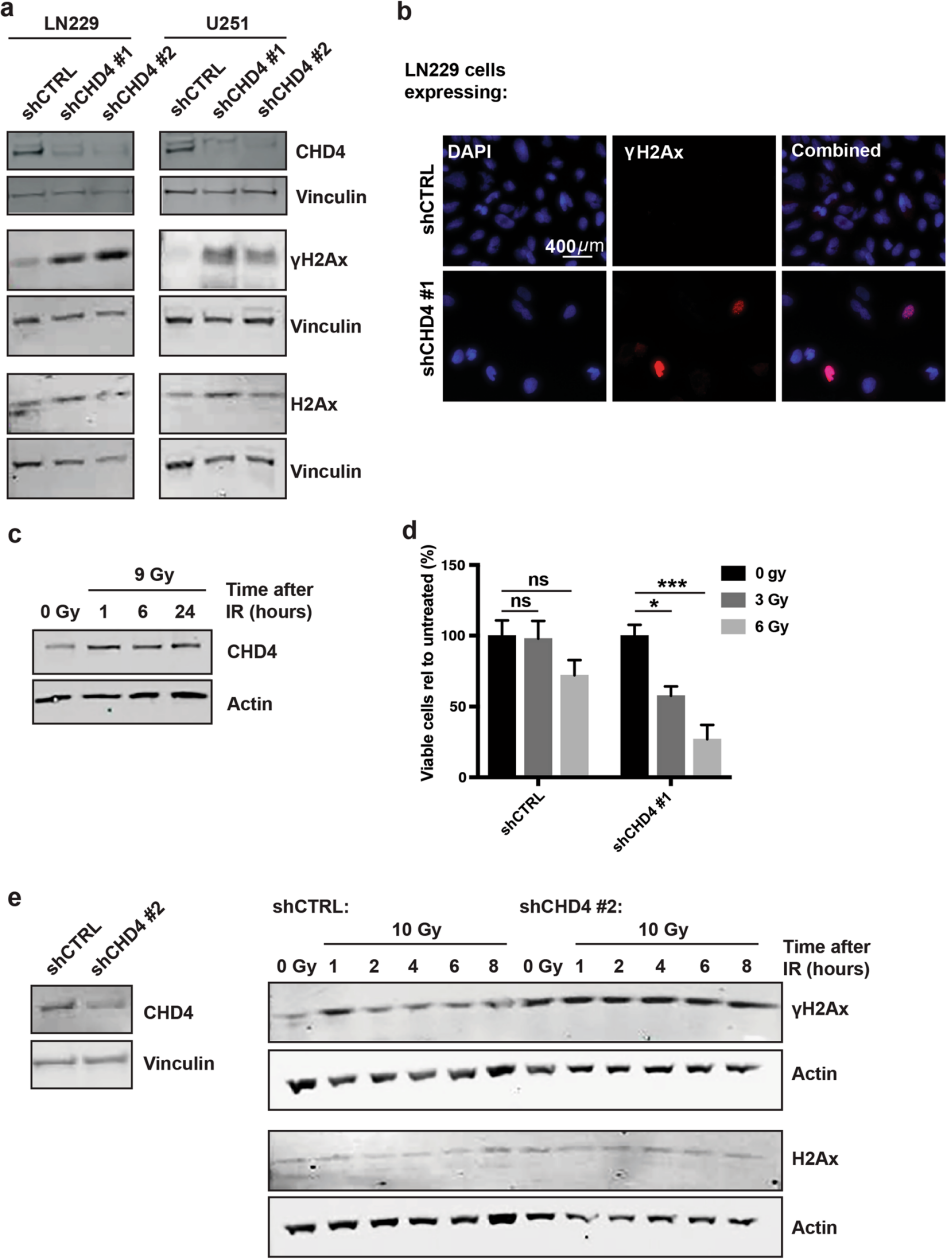
CHD4 suppression causes increased γH2Ax, and prolongs time to repair after ionizing radiation (IR). (**a**) Immunoblot for γH2Ax after CHD4 suppression in LN229 and U251 cells. Full-length blots are presented in Supplementary Fig. 3. (**b**) Immunofluorescent staining of LN229 cells with DAPI (blue), γH2Ax (red), and combined after expression of shCTRL or shCHD4#1. (**c**) Immunoblot for CHD4 expression over time in LN229 cells exposed to 9 Gy radiation. Full-length blots are presented in Supplementary Fig. 3. (**d**) Cell number of LN229 cells expressing shCTRL or shCHD4#1 3 days after exposure to either 0 Gy, 3 Gy, or 6 Gy radiation. Cell number is expressed as a percentage relative to 0 Gy (shCTRL cells: 0 Gy vs. 3 Gy, p = < 0.99999; 0 Gy vs. 6 Gy, p = 0.141; shCHD4#1 cells: 0 Gy vs. 3 Gy, p = 0.0144; 0 Gy vs. 6 Gy, p = 0.0001). (**e**) Immunoblots for γH2Ax in LN229 cells expressing shCTRL or shCHD4 #2 after either no radiation (0 Gy) or 1, 2, 4, 6 or 8 hours after exposure to 10 Gy radiation. The corresponding immunoblot for CHD4 expression after shCTRL or shCHD4#2 is also shown. Full-length blots are presented in Supplementary Fig. 3. Data in (**d**) is shown as mean ± SEM; *p ≤ 0.05; ***p ≤ 0.0001. All data is representative of three independent experiments (with three technical replicates each).

**Figure 4:**
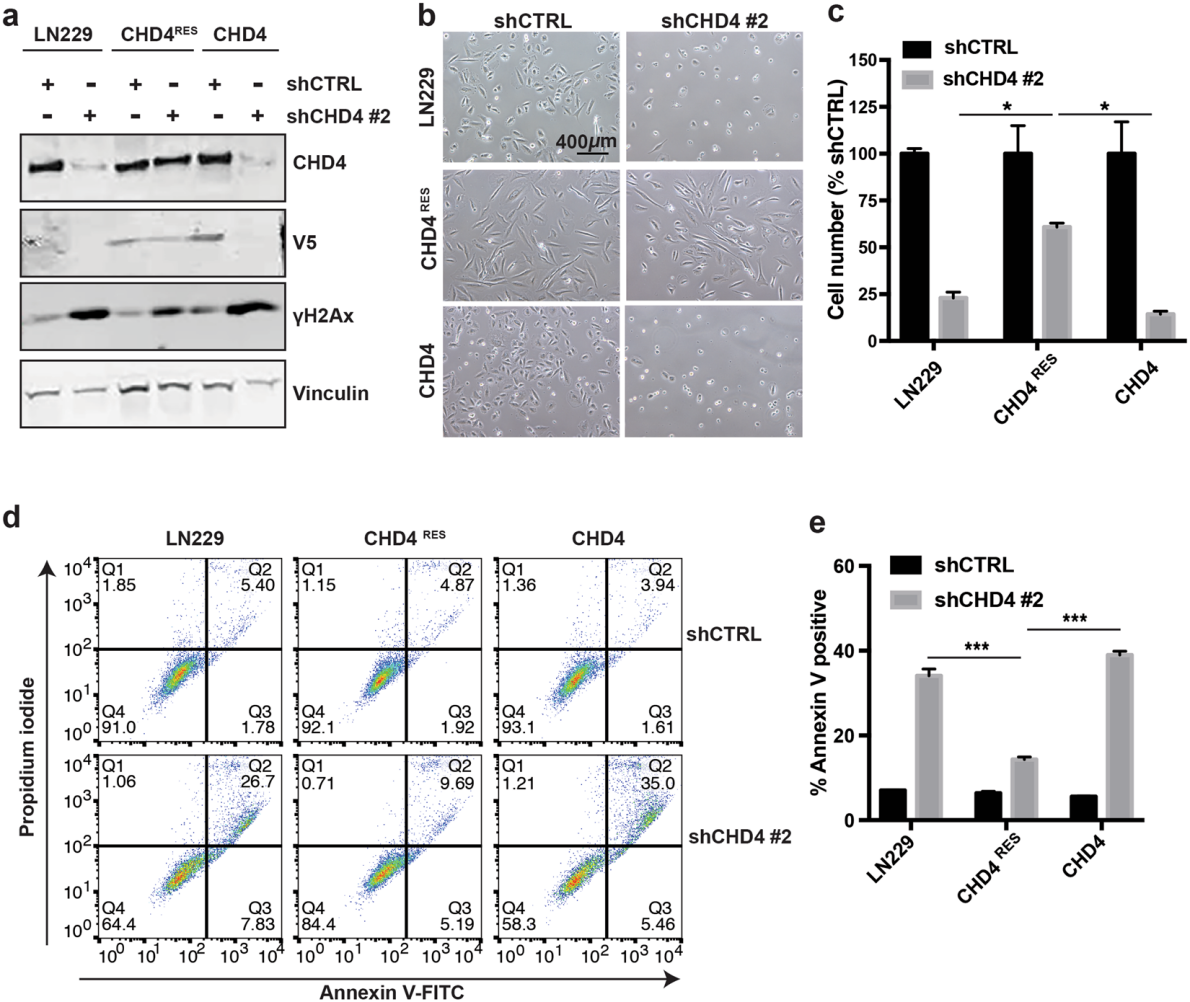
Expression of an shCHD4#1 resistant CHD4 construct partially rescues cell viability and increased γH2Ax caused by CHD4 suppression. (**a**) Immunoblot for endogenous CHD4 and overexpressed v5-tagged shRNA-resistant CHD4 (CHD4^RES^) or wild type (CHD4^WT^), and γH2Ax in LN229 cells. Full-length blots are presented in Supplementary Fig. 3. (**b**) Brightfield images of indicated cells. (**c**) Cell number (percent relative to shCTRL) after expression of shCTRL or shCHD4 #2 in LN229, CHD4^RES^, and CHD4 cells. (LN229 shCHD4 #2 vs. CHD4^RES^ shCHD4 #2, *p* = 0.0374; CHD4^RES^ shCHD4 #2 vs. CHD4 shCHD4 #2, *p* = 0.0118). (**d**) Annexin V and PI labelling of the indicated cells after expression of shCTRL and shCHD4 #2. (**e**) Quantification of the Annexin V positive cells from (**e**). (LN229 shCHD4 #2 vs. CHD4^RES^ shCHD4 #2, *p* < 0.0001; CHD4^RES^ shCHD4 #2 vs. CHD4 shCHD4 #2, *p* < 0.0001). Data in (**d**,**f**) is shown as mean ± SEM; *p ≤ 0.05; ***p ≤ 0.0001. Full-length blots are presented in Supplementary Fig. 3. Data in (**c**,**e**) is shown as mean ± SEM; *p ≤ 0.05; ***p ≤ 0.0001. All data is representative of two independent experiments (with three technical replicates each).

Previous studies have shown that CHD4 has a role in the DNA damage response (DDR)^8,11,17^. We therefore investigated whether the increased cell death was a consequence of defective DDR. We found that even in the absence of external DNA damage, CHD4 suppression caused an increase in phosphorylated H2Ax (γH2Ax) in GBM cell lines (Fig. 3a,b) and tumour initiating cells (Supplementary Fig. 1), indicating an increase in DNA DSBs. To confirm that the observed results were not due to off-target effects of the shRNAs, we generated an shRNA-resistant CHD4 allele (CHD4^RES^). The resistant allele partially rescued CHD4 protein and γH2Ax levels (Fig. 4a), and partially rescued cell viability (Fig. 4b,c) and prevented apoptosis (Fig. 4d,e). To further investigate the role of CHD4 in the DDR in GBM cells, we exposed LN229 cells to radiation and observed elevated CHD4 expression within 1 hour (Fig. 3c). We also found that cell viability was significantly decreased in CHD4-suppressed LN229 cells after exposure to radiation, whereas control cell viability was not significantly changed (Fig. 3d), indicating that CHD4 suppression sensitises cells to radiation-induced DNA damage. Further, to assess the role of CHD4 in the setting of exogenous DNA damage, we measured γH2Ax levels over time after irradiation. As expected, control cells showed an initial increase in γH2Ax, followed by resolution to endogenous levels by 2 hours after irradiation. CHD4-depleted LN229 cells did not show an increase in γH2Ax after irradiation (Fig. 3e) compared to the non-irradiated CHD4-depleted cells. This suggests that H2Ax is already maximally activated in cells lacking CHD4. We had similar results in tumour initiating cells. CHD4 suppression resulted in higher basal levels of γH2Ax and sustained γH2Ax after irradiation (Supplementary Fig. 1). These results indicate that under basal conditions, CHD4 is required for DSB repairs, and suggest that in the absence of CHD4, there is a deficit in repair and resolution of γH2Ax after radiation exposure.

### CHD4 positively regulates HR activity and RAD51 expression

**Figure 5:**
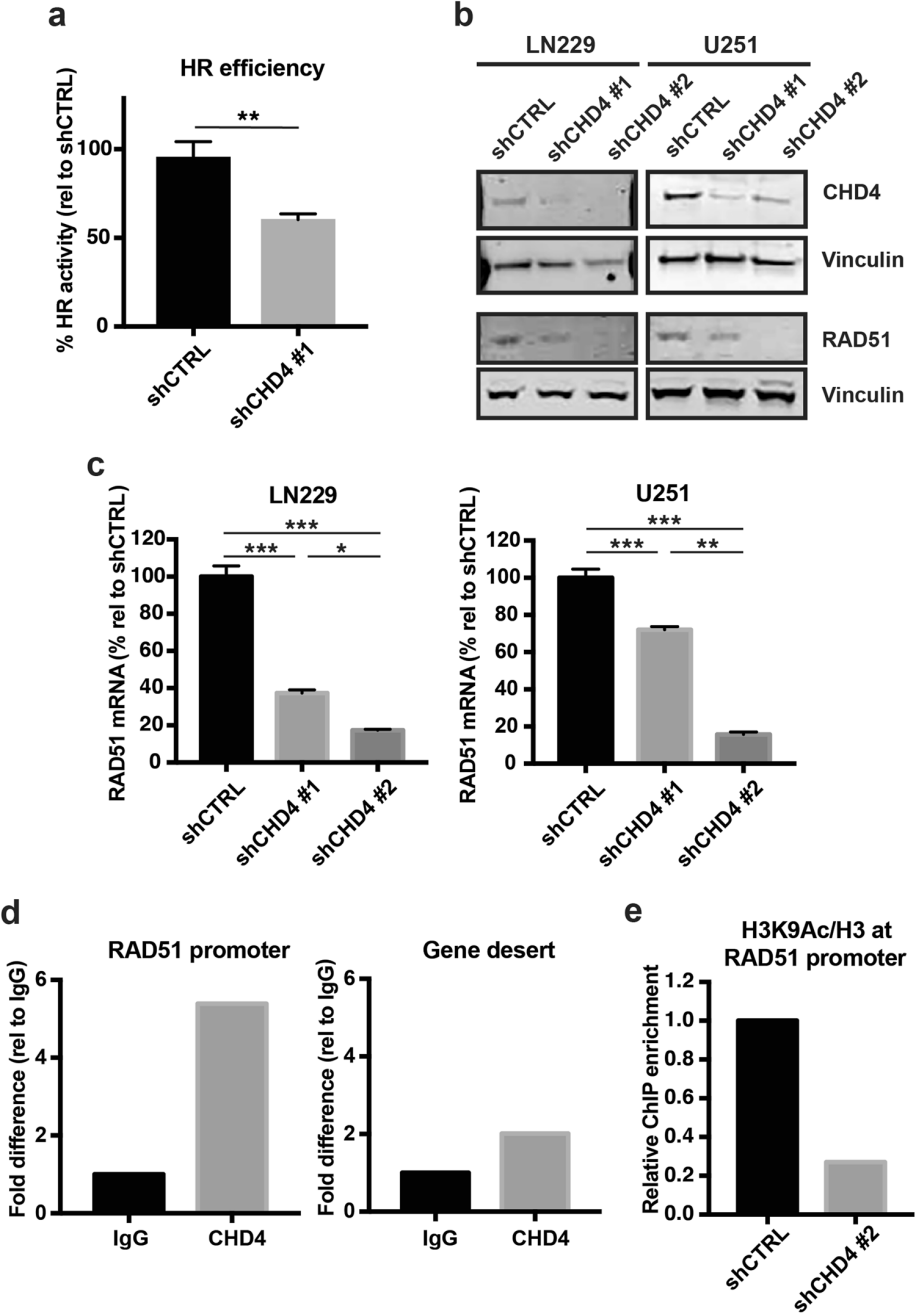
CHD4 suppression results in decreased HR efficiency, and decreased RAD51 protein, mRNA, and promoter activity. (**a**) Homologous recombination (HR) activity assay for LN229 cells expressing shCTRL or shCHD4#1 (p = 0.0023). Data is representative of three independent experiments with three technical replicates. (**b**) Immunoblots for CHD4 and RAD51 in LN229 and U251 cells expressing shCTRL, or two shRNAs targeting CHD4. Full-length blots are presented in Supplementary Fig. 3. (**c**) qRT-PCR analysis of RAD51 mRNA expression in LN229 and U251 cells expressing shCTRL, or two shRNAs targeting CHD4. All values were normalized to tubulin mRNA and are shown as percent expression relative to shCTRL (LN229: shCTRL vs. shCHD4 #1, *p* < 0.0001, shCTRL vs. shCHD4 #2, *p* < 0.0001; U251: shCTRL vs. shCHD4 #1, *p* = 0.0013, shCTRL vs. shCHD4 #2, *p* = < 0.0001). (**d**) ChIP qPCR analysis of CHD4 occupancy at the RAD51 promoter or a gene desert (as a control). Data shown as a fold difference compared with IgG, and is representative of two independent experiments. (**e**) ChIP-qPCR analysis of H3K9Ac occupancy at the RAD51 promoter or gene desert control after transduction of shCTRL or shCHD4#2. Data is shown as fold difference relative to IgG, and is normalized to H3 occupancy on the RAD51 promoter and gene desert, and is representative of two independent experiments (with three technical replicates each). Data in (**a**,**c**) shown as mean ± SEM; *p ≤ 0.05; **p ≤ 0.001; ***p ≤ 0.0001.

To determine why CHD4 silencing resulted in increased γH2Ax in GBM cells, we measured homologous recombination (HR) efficiency after CHD4 suppression. HR is a major DNA repair mechanism that is important in DSB repair. In comparison with the control, HR efficiency in cells with CHD4 suppression was significantly decreased (Fig. 5a). To further investigate this decreased HR efficiency, we measured RAD51 expression. RAD51 is an essential component of HR-mediated DNA damage repair; RAD51 promotes single strand invasion during HR^28,29^. RAD51 is overexpressed in multiple cancers, correlates with poor prognosis^30^, and is associated with radioresistance in GBM^31^. When we depleted CHD4, there was decreased RAD51 protein and mRNA in GBM cell lines (Fig. 5b,c) and tumour initiating cells (Supplementary Fig. 1). Since RAD51 expression is altered during different phases of the cell cycle^32,33^, we performed cell cycle analysis using PI staining and flow cytometry. We did not observe a significant change in cell cycle profile or increased p21 expression in either cell line (Supplementary Fig. 2), indicating that the decreased RAD51 expression is not a response to alterations in cell cycle phase. Therefore, these results demonstrate that CHD4 regulates RAD51 protein and mRNA expression in GBM cells.

### CHD4 binds to the RAD51 promoter and promotes an active chromatin state

CHD4 is a member of complexes that transcriptionally regulate genes^6,34^. We therefore performed chromatin immunoprecipitation followed by quantitative PCR (ChIP qPCR) to determine whether CHD4 binds the RAD51 promoter. We found that CHD4 is enriched at the RAD51 promoter when compared with IgG isotype and gene desert controls (Fig. 5d). To determine whether CHD4 localization at the RAD51 promoter causes a more permissive chromatin environment there, we then performed ChIP for acetylated H3 at lysine 9 (H3K9Ac), an active histone mark, on the RAD51 promoter. We found that H3K9Ac levels consequently decreased at the RAD51 promoter after CHD4 depletion (Fig. 5e) compared to controls, suggesting that CHD4 localization to the RAD51 promoter produces a permissive and active chromatin state that in turn may promote expression of RAD51.

## Discussion

A longstanding challenge in neuro-oncology has been resistance to DNA damaging therapies. Our work advances the understanding of the factors that promote this resistance, and points to one possible strategy to enhance cell death. We show that CHD4 expression correlates with poor patient outcomes, and is a specific vulnerability in brain tumour cells. King and colleagues recently demonstrated that RAD51 expression contributes to resistance of GBM cells to radiation^31^. Here, we demonstrate that CHD4 drives this increased expression.

CHD4 is already understood to play a role in some aspects of DNA damage and resistance. CHD4 recruits DDR proteins to sites of double strand breaks and mounts a response to DNA damage in solid and hematologic cancers^8,23,35^. In addition, GBM tumour initiating cells, which are highly resistant to DNA damaging agents^36,37^, overexpress RAD51^31^; we previously showed that CHD4 is required for this cancer stem cell-like state^16^. Our findings are also consistent with recent work by Kitange and colleagues, who demonstrated that NuRD member RBBP4 drives RAD51 transcription by interacting with p300/CPB co-activator complex to generate a permissive chromatin environment^38^. Given that CHD4 co-immunoprecipitates with p300^20^, and p300 presence activates RAD51 transcription^39^, our observations contribute to an emerging model of CHD4 and NuRD working in complex with p300 to drive RAD51 transcription.

Our study suggests that CHD4 is required for the survival of GBM cell lines, and its suppression has only minor effects on normal human astrocytes. We hypothesized that CHD4 loss causes cell death because it renders the cells ineffective in DNA damage repair. We found that even in unstressed conditions, CHD4 depletion increased γH2Ax levels. While others have shown that CHD4 loss in other cancer contexts causes a delay in DNA damage repair after exposure to DNA damaging agents^8,15,19,23,35^; it is rarer that its suppression increases γH2Ax expression in the absence of exogenous damage. Such changes have only been reported in ovarian and breast cancer cell lines with intact BRCA2^24^. We did not observe increased γH2Ax in unstressed non-transformed astrocytes after CHD4 suppression, however, others have reported such findings in primary human fibroblasts^40^. Given that CHD4 dynamically moves the position of nucleosomes on DNA, it is possible that the loss of CHD4 in GBM cells causes global chromatin relaxation. This is the case in AML^23^, HeLa^40^ and Ramos^41^ cells, where loss of CHD4 causes increased global H3K9Ac levels, which is indicative of a more relaxed chromatin state. This more relaxed chromatin state might thereby provide DNA less protection against environmental DNA damage^42,43^. Interestingly, we found that exposing CHD4-suppressed GBM cells to radiation did not result in a further increase in γH2Ax expression atop that seen with CHD4 suppression alone. This suggests that CHD4 suppression maximally activates γH2Ax in our system. In contrast, exposing CHD4-suppressed GBM tumour initiating cells did result in an increase in γH2Ax expression, which was not resolved to endogenous levels after 24 hours. Clinically, this suggests that pharmacologic methods to suppress CHD4 activity might synergize with radiation, and perhaps even enable a reduction in radiation and its associated toxicities. This is consistent with other studies that have shown that inhibition of some HR proteins can sensitize GBM cells to radiation^31,44,45^.

In summary, CHD4 is overexpressed in GBM and associated with worse outcome. In GBM cells, its suppression enhances DNA damage and decreases RAD51 expression. Therefore, reducing CHD4 activity or level may be one way to enhance the effects of radiation and temozolomide. Given the minimal effects in non-transformed astrocytes, such a strategy might leverage a therapeutic window. Lastly, since CHD4 loss also causes differentiation of GBM tumour initiating cells^16^, it may play multiple important roles in GBM pathogenesis and resistance to therapy.

## Materials and Methods

### Cell lines and cell culture

LN229 and U251 cells were maintained in DMEM with high glucose (Gibco, cat#11965-092) Supplemented with 10% foetal bovine serum (Sigma, cat#F2442) and 1% penicillin/streptomycin (Gibco, cat#15140-122). Normal human astrocytes (NHA) were purchased from Sciencell and maintained in Astrocyte Medium Supplemented with 2% foetal bovine serum, 1% astrocyte growth Supplement and 1% penicillin-streptomycin (Sciencell Research Laboratories, cat#1801). 0308^46^, TS667, BT112^47^ and TS665 patient-derived cell lines were maintained in NBE medium: Neurobasal medium (Gibco) containing N-2 (Gibco) and B-27 without vitamin A (Gibco) Supplements, EGF (Peprotech), bFGF (Peprotech), L-glutamine (Gibco), and penicillin-streptomycin (Gibco) as described^46^. All cells were maintained at 37 C in a humidified incubator with 5% CO_2_.

### DNA Constructs

*shRNA constructs:* Lentiviral shRNA constructs were obtained from The RNAi Consortium (TRC) of the Broad Institute^48^. The following shRNA clones were used: shCTRL is an shRNA targeting LACZ: TRCN0000072235, 5′-CCGG-CCGTCATAGCGATAA CGAGTT-CTCGAG-AACTCGTTATCGCTATGAC GG- TTTTTG-3′; target sequence: CCGTCATAGCGATAACGAGTT; shCHD4#1: TRCN0000021363, 5′-CCGG-GCGGGAGTTCAGTACCAATAA -CTCGAG-TTATTG GTACTGAACTCCCGC- TTTTT-3′ target sequence: GCGGGAGTTCAGTACCAATAA; shCHD4#2: TRCN0000021361, 5′-CCGG-GCTGCTGAC ATCCTATGAATT-CTCGAG-AATTCATAGG ATGTCAGCAGC- TTTTT-3′, target sequence: GCTGCTGACATCCTATGAATT. *Overexpression construct:* pLX304 was a gift from David Root (Addgene plasmid # 25890)^49^. pDONR221 CHD4 was obtained from the Harvard Plasmid Database (clone ID: HsCD00080095). pLX304 CHD4 (v5-tagged) plasmid was made by performing a Gateway cloning reaction using LR Clonase II Plus enzyme mix (Invitrogen, Cat# 12538-120). shCHD4#2-resistant pLX304 CHD4 plasmid was made by creating 4 silent mutations in bases within the shCHD4#2 target sequence using the Quickchange mutagenesis kit as per manufacturer’s instructions. The following primer sequences were used for mutagenesis sequentially:

SET #1 – F: 5′-GGTGATCAATTCATAGGACGTCAGCAGCACATGGAAT-3′

R: 5′-ATTCCATGTGCTGCTGACGTCCTATGAATTGATCACC-3′

SET #2 – F: 5′-TGTCAATGGTGATCAATTCATACGACGTTAGTAGCACATG GAATTTCACAGATG-3′; R: 5′-CATCTGTGAAATTCCATGTGCTACTAACGTCGT ATGAATTGATCACCATTGACA-3′

SET #3 – F: 5′-TGTGAAATTCCATGTGCTACTAACGTCGTACGAGCTGATCAC CATTGACATG-3′; R: 5′-CATGTCAATGGTGATCAGCTCGTACGACGTTAGTAG CACATGGAATTTCACA-3′

SET #4 – F: 5′-CTTACGAGCTGGATCACCATTGACATGGC-3′; R: 5′-ACGTG AGGAGCACATGGAATTTCACAGATGC-3′.

### Immunoblot

For protein extraction, cells were lysed with RIPA buffer (10 mM Tris-HCl, 1 mM EDTA, 0.5 mM EDTA, 1% Triton X-100, 0.1% sodium deoxycholate, 0.1% SDS, 140 mM NaCl) Supplemented with 1 X Complete EDTA-free protease and phosphatase inhibitor (Roche, cat#04693132001). Protein concentration was determined using Pierce BCA protein assay kit (Thermo Fisher Scientific, cat#23225). An equal amount of protein was loaded per lane into SDS-PAGE gels (BOLT 4–12% Bis-Tris gels, cat#NW04120BOX; or NuPage 3–8% Tris-Acetate gels, cat#EA0378BOX), followed by transfer onto 0.45 µM nitrocellulose membranes (Amersham, 10600008). Membranes were blocked with Odyssey blocking buffer (LICOR, cat#927-40000), followed by overnight incubation with the primary antibody diluted in Odyssey blocking buffer. After washing 3X in PBS, membranes were incubated with secondary infrared antibodies diluted in blocking buffer, washed a further 3X in PBS, then imaged using the LICOR Odyssey Infrared Imaging System. Primary antibodies: anti-Actin (goat), 1:2000, Santa Cruz, cat#sc-1616; anti-Vinculin (Rabbit), 1:2000, Abcam, cat#ab129002; anti-CHD4 (Rabbit), 1:250, Abcam, cat#ab72418; anti- γH2Ax (Rabbit), 1:1000, Abcam, cat#ab11174; anti-H2AX (Rabbit), 1:250, Cell Signaling, cat#2595; anti-V5 (Rabbit), Millipore, cat#AB3792; anti-RAD51 (Rabbit), Cell Signaling, cat#8875. Secondary antibodies: IRDye 680 LT (rabbit), 1:10,000, LICOR, cat#926-68073; IRDye 680 (mouse), 1:10,000, LICOR, cat#926-68072; IRDye 800 (goat), 1:10,000, LICOR, cat#926-32214.

### Cell viability assay

Cells were seeded at a density of 1 × 10^5^ cells per well in a 6-well tissue-culture treated plate and transduced overnight with lentiviral shRNAs Supplemented with polybrene (1 µg/mL; Santa Cruz, cat#sc-134220). Media was then removed and replaced with fresh media containing puromycin (2 µg/mL, Gold Bio, cat#P-600-1). After two days of selection, cells were counted and 1 × 10^5^ cells/well were plated in a 6-well plate, in triplicate. Three days later, cells were counted with trypan blue exclusion.

### Apoptosis analysis

Apoptosis analysis was performed using the Annexin V-FITC + PI Apoptosis Detection Kit (Leinco Technologies, cat#A432). Briefly, cells were transduced with shRNAs, selected with puromycin for 2 days, and then an equal number of cells were re-plated in triplicate. Three days later, floating cells and adherent cells were combined and counted. Cells were re-suspended in 1 X Binding buffer to a concentration of 1 × 10^6^ cells/mL, and 10 × 10^5^ cells were transferred to FACS tubes (Falcon, cat#352235). 5 µl of Annexin V-FITC and 5 µl of PI were added per tube, along with single-stain and unstained controls. Cells were incubated in a 37 C incubator for 15 mins, and then analysed using flow cytometry (Becton Dickinson FACS Calibur). Data analysis was performed using FlowJo version 10.

### Cell cycle analysis

LN229 and U251 cells, after shRNA transduction and selection with puromycin, were re-plated at a density of 1 × 10^5^ cells per well of a 6-well plate in triplicate. After 3 days, cells were washed with PBS, trypsinized and counted. 2 × 10^5^ cells were fixed in 70% ice cold ethanol for at least 30 mins, washed 3 X with ice cold PBS, the supernatant removed, and then resuspended in PI staining solution (0.1 mg/mL PI, 0.1% Triton X, 2.5 µg/mL RNAse A) and placed in FACS tubes (Falcon, cat#352235). Cells were incubated at 37 C for 15 mins, and then analysed using flow cytometry (Becton Dickinson FACS Calibur). Data analysis was performed using FlowJo version 10.

### Immunofluorescence

1 × 10^4^ LN229 cells expressing either shCTRL or shCHD4#1 were plated on Falcon 8-well culture slides (Corning, cat# 354118). Cells were washed 3 X in PBS, followed by a 5-minute incubation in PBST (PBS + 0.05% tween) to permeabilise cells. Cells were washed 3 X in PBS, then fixed for 20 mins in 4% formalin. After a further 3 X PBS washes, cells were blocked in Odyssey blocking buffer (LICOR, cat#927-40000) for 30 mins, then incubated with anti- γH2Ax (Rabbit) antibody, (1:500, Abcam, cat#ab11174) diluted in Odyssey blocking buffer for 1 hr. Cells were then incubated with anti-rabbit alexa fluor 568 (1:1000, Invitrogen, cat#A-11011) for 1 hr, washed 3 X in PBS, then incubated with 1ug/ml DAPI (Invitrogen, cat# D1306) for 10 mins. Slides were mounted using Prolong Diamond mounting medium (Invitrogen, cat#P3670) and imaged using a Nikon Eclipse 80i microscope.

### Radiation treatment

CHD4 protein expression in response to radiation: LN229 cells were exposed to 9 Gy radiation and protein was isolated 1 hr, 6 hr, and 24 hrs after treatment. Immunoblots for CHD4 (anti-CHD4 (Rabbit), 1:250, Abcam, cat#ab72418) and Vinculin (anti-Vinculin (Rabbit), 1:2000, Abcam, cat#ab129002) were performed as previously described. CHD4 suppression and cell growth after radiation: shRNA-transduced LN229 cells were selected with puromycin for two days, counted and re-plated at a density of 1 × 10^5^ cells per well of a 6-well plate in triplicate. After three days, cells were either left untreated (0 Gy) or irradiated with 3 Gy or 6 Gy. Cells were then counted after a further 3 days. γH2Ax expression after CHD4 suppression and radiation treatment: shRNA-transduced LN229 cells were selected with puromycin for two days, counted and re-plated at a density of 1 × 10^5^ cells per well of a 6-well plate in triplicate. After three days, cells were either left untreated (0 Gy) or irradiated with 10 Gy or 3 Gy. Protein was isolated at 1 hr, 2 hr, 4 hr, 6 hr and 8 hr (for LN229 cells) or 1.5 hr, 3 hr, 5 hr, 8 hr, 12 hr or 24 hr (for TS667 cells) after exposure to radiation. All radiation experiments were performed using an X-RAD 320 biological irradiator (Precision X-ray Inc.).

### Homologous Recombination Assay

A homologous recombination assay was performed according to the manufacturer’s instructions (Norgen Biotek Corp., cat#35600). Briefly, LN229 cells expressing either shCTRL or shCHD4#1 were co-transfected with dl-1 and dl-2 plasmids (or negative and positive control plasmids), and total genomic DNA was isolated 48 hours later using the DNeasy mini kit (Qiagen, cat#69504). A qPCR reaction was performed using the supplied primers to determine HR efficiency. If HR is perturbed, the dl-1 and dl-2 plasmids will not recombine to produce a PCR product; therefore, the amount of PCR product is directly correlated with HR efficiency.

### RNA extraction, cDNA synthesis, and quantitative RT-PCR

RNA was extracted using the RNeasy mini kit (QIAGEN, cat#74104) with an on-column RNase-free DNase digestion (QIAGEN, cat#79254). cDNA synthesis was performed using the Superscript III first-strand synthesis kit (Invitrogen, cat#18080-051). For qRT-PCR, 50 ng of cDNA was added to SYBR Power master mix (Applied Biosystems, cat#A25742) and the relevant forward and reverse primers. qRT-PCR was performed using CFX96 Real-Time PCR machine (Biorad) under the following conditions: pre-heating step for 10 mins at 95 C, then 40 cycles of 95 C for 15 sec and 60 C for 1 min, and an ended with a melt curve analysis. Results were analysed using the 2^−ΔΔct^ method. Primer sequences: RAD51_F: 5′-CAACCCATTTCACGGTTAGAG C-3′; RAD51_R: 5′-TTCTTTGGCGCATAGGCAACA-3′; TUBULIN_F: 5′-TGGAC TCTGTTCGCTCAGGT-3′; TUBULIN_R: 5′-TGCCTCCTTCCGTACCACAT-3′.

### Chromatin immunoprecipitation (ChIP) and quantitative PCR

1 × 10^7^ cells were crosslinked by adding 1% paraformaldehyde (Electron Microscopy Sciences, cat#15710) to cells in culture media for 10 mins at room temperature, while rotating. The reaction was quenched with glycine to a final concentration of 0.125 M, for 5 mins. Cells were centrifuged, the supernatant discarded, and washed twice with cold PBS. Cell pellets were frozen at −80C until required. For immunoprecipitation, 50 µL of Protein G Dynabeads (Invitrogen, cat#100040) were washed 3X in PBS + 0.1% Tween 20, then incubated with 8 µg of the required primary antibodies or isotype control for 6 hours at 4 C. Primary antibodies: anti-CHD4 (Rabbit), Abcam, cat#ab72418; anti-H3 (Rabbit), Cell Signaling, cat#9649; anti-H3K9Ac (Rabbit), Cell Signaling, cat#4499; anti-IgG (Rabbit), Millipore, cat#12370. In parallel, crosslinked cells were then thawed, lysed for 20 mins in SDS lysis buffer (1% SDS, 10 mM EDTA, 50 mM Tris-HCl), then 400–1000 bp fragments of chromatin were produced by sonication in a water bath sonicator (4 C, Misonix ultrasound liquid processor) for 13 cycles of 30 sec on, 30 sec off at 65 Amps. Lysates were pre-cleared for 1–2 hrs with beads, and 10% of lysate was saved. Antibody-incubated beads were washed 3X, and lysate was diluted 1:2 with ChIP dilution buffer (0.01% SDS, 1.1% Triton X, 1.2 mM EDTA, 16.7 mM Tris-HCl, 167 mM NaCl), then incubated with the beads overnight at 4 C with rotation. Beads were then washed for 3 mins in low salt wash buffer (0.1% SDS, 1% Triton X, 2 mM EDTA, 20 mM Tris-HCl, 150 mM NaCl), high salt wash buffer (0.1% SDS, 1% Triton X, 2 mM EDTA, 20 mM Tris-HCl, 500 mM NaCl), LiCl wash buffer (0.25 M LiCl, 1% NP40, 1% sodium deoxycholate, 1 mM EDTA, 10 mM Tris-HCl), and twice in TE buffer. DNA-protein complexes were eluted from the beads by adding 125 µl elution buffer (1% SDS, 0.1 M NaHCO_3_) for 15 mins at room temperature twice. Input samples were diluted to 250 µl with ChIP dilution buffer. DNA-protein complexes were de-crosslinked by adding 10 µl 5 M NaCl to the samples and incubating at 65 C overnight. For DNA isolation, proteinase K solution (1 µl proteinase K, 5 µl 0.5 M EDTA, and 10 µl 1 M Tris-HCl) was added to each sample and incubated for 2 hours at 45 C, then purified using a QIAquick PCR purification column (QIAGEN, cat#28106). Quantitative PCR was then performed with input and immunoprecipitated DNA using primers directed to the RAD51 promoter (F: 5′-CCCCCGGCATAAAGTTTGA-3′; R: 5′-GCTTTCAGAATTCCCGCCA-3′ ^38,39^), or IGX1A gene desert (QIAGEN, cat#GPH100001C(-)01 A) and Power SYBR according to manufacturer’s instructions, and analysed using a CFX96 Real-Time PCR machine.

### Patient data analysis

The Cancer Genome Atlas (TCGA)^25^ and Rembrandt^26^ patient data and gene expression datasets were analysed using GlioVis data portal^27^. CHD4 mRNA expression data was downloaded from GlioVis data portal and graphs and pairwise t-tests with Bonferroni multiple hypothesis correction were performed using Graphpad Prism version 7.0a.

### Statistical analysis

ChIP PCR experiments were performed in triplicate two independent times. All other observations were made in triplicate in at least three independent experiments. Quantitative data is expressed as mean ± SEM. Datasets with two groups were analysed using unpaired t-tests; datasets with three groups were analysed using one-way ANOVA and post-hoc Tukey test. Survival analysis was performed using the Kaplan-Meier method and analysed by log-rank test.

## Supplementary information

Supplementary figures

## Data Availability

The data that supports the findings of this study will be made available by the corresponding author upon request.

## References

[1] Bleeker FE, Molenaar RJ, Leenstra S (2012). Recent advances in the molecular understanding of glioblastoma. J Neurooncol.

[2] Stupp R (2005). Radiotherapy plus concomitant and adjuvant temozolomide for glioblastoma. N. Engl. J. Med..

[3] Sulman EP (2017). Radiation Therapy for Glioblastoma: American Society of Clinical Oncology Clinical Practice Guideline Endorsement of the American Society for Radiation Oncology Guideline. J Clin Oncol.

[4] Stupp R (2009). Effects of radiotherapy with concomitant and adjuvant temozolomide versus radiotherapy alone on survival in glioblastoma in a randomised phase III study: 5-year analysis of the EORTC-NCIC trial. Lancet Oncol.

[5] Watson AA (2012). ThePHD and Chromo Domains Regulate the ATPase Activity of the Human Chromatin Remodeler CHD4. J Mol Biol..

[6] Denslow SA, Wade PA (2007). The human Mi-2/NuRD complex and gene regulation. Oncogene.

[7] O’Shaughnessy A, Hendrich B (2013). CHD4 in the DNA-damage response and cell cycle progression: not so NuRDy now. Biochm. Soc. Trans..

[8] Polo SE, Kaidi A, Baskcomb L, Galanty Y, Jackson SP (2010). Regulation of DNA‐damage responses and cell‐cycle progression by the chromatin remodelling factor CHD4. EMBO J..

[9] O’Shaughnessy-Kirwan A, Signolet J, Costello I, Gharbi S, Hendrich B (2015). Constraint of gene expression by the chromatin remodelling protein CHD4 facilitates lineage specification. Development.

[10] Ingram KG, Curtis CD, Silasi-Mansat R, Lupu F, Griffin CT (2013). The NuRD Chromatin-Remodeling Enzyme CHD4 Promotes Embryonic Vascular Integrity by Transcriptionally Regulating Extracellular Matrix Proteolysis. PLOS GENET.

[11] Lai AY, Wade PA (2011). Cancer biology and NuRD: a multifaceted chromatin remodelling complex. Nat. Rev. Cancer.

[12] Le Gallo M (2012). Exome sequencing of serous endometrial tumors identifies recurrent somatic mutations in chromatin-remodeling and ubiquitin ligase complex genes. Nature genet.

[13] Xu N, Liu F, Zhou J, Bai C (2016). CHD4 is associated with poor prognosis of non-small cell lung cancer patients through promoting tumor cell proliferation. Eur Respir J.

[14] Nio K (2015). Defeating EpCAM+liver cancer stem cells by targeting chromatin remodeling enzyme CHD4 in human hepatocellular carcinoma. J. Hepatol..

[15] Xia L (2017). CHD4 Has Oncogenic Functions in Initiating and Maintaining Epigenetic Suppression of Multiple Tumor Suppressor Genes. Cancer Cell.

[16] Chudnovsky Y (2014). ZFHX4 interacts with the NuRD core member CHD4 and regulates the glioblastoma tumor-initiating cell state. Cell Rep.

[17] Smeenk G (2010). The NuRD chromatin–remodeling complex regulates signaling and repair of DNA damage. J Cell Biol.

[18] Chou DM (2010). A chromatin localization screen reveals poly (ADP ribose)-regulated recruitment of the repressive polycomb and NuRD complexes to sites of DNA damage. Proc Natl Acad Sci USA.

[19] Larsen DH (2010). The chromatin-remodeling factor CHD4 coordinates signaling and repair after DNA damage. J Cell Biol.

[20] Qi W (2016). Acetyltransferase p300 collaborates with chromodomain helicase DNA-binding protein 4 (CHD4) to facilitate DNA double-strand break repair. Mutagenesis.

[21] Urquhart AJ, Gatei M, Richard DJ, Khanna KK (2011). ATM mediated phosphorylation of CHD4 contributes to genome maintenance. Genome Integr.

[22] Schmidt DR, Schreiber SL (1999). Molecular association between ATR and two components of the nucleosome remodeling and deacetylating complex, HDAC2 and CHD4. Biochem..

[23] Sperlazza J (2015). Depletion of the chromatin remodeler CHD4 sensitizes AML blasts to genotoxic agents and reduces tumor formation. Blood.

[24] Guillemette S (2015). Resistance to therapy in BRCA2 mutant cells due to loss of the nucleosome remodeling factor CHD4. Genes Dev..

[25] Cancer Genome Atlas Research Network (2008). Comprehensive genomic characterization defines human glioblastoma genes and core pathways. Nature.

[26] Madhavan S (2009). Rembrandt: helping personalized medicine become a reality through integrative translational research. Mol Cancer Res.

[27] Bowman RL, Wang Q, Carro A, Verhaak RGW, Squatrito M (2017). GlioVis data portal for visualization and analysis of brain tumor expression datasets. Neuro Oncol.

[28] Sung P, Krejci L, Van Komen S, Sehorn MG (2003). Rad51 Recombinase and Recombination Mediators. J Biol Chem.

[29] Baumann P, West SC (1998). Role of the human RAD51 protein in homologous recombination and double-stranded-break repair. Trends Biochem Sci.

[30] Klein HL (2008). The Consequences of Rad51 Overexpression for Normal and TumorCells. DNA repair.

[31] King HO (2017). RAD51 Is a Selective DNA Repair Target to Radiosensitize Glioma Stem Cells. Stem Cell Reports.

[32] Flygare J, Benson F, Hellgren D (1996). Expression of the human RAD51 gene during the cell cycle in primary human peripheral blood lymphocytes. Biochim Biophys Acta.

[33] Chen F (1997). Cell cycle-dependent protein expression of mammalian homologs of yeast DNA double-strand break repair genes Rad51 and Rad52. Mutat Res.

[34] Hosokawa H (2013). Functionally distinct Gata3/Chd4 complexes coordinately establish T helper 2 (Th2) cell identity. Proc Natl Acad Sci USA.

[35] Pan M-R (2012). Chromodomain helicase DNA-binding protein 4 (CHD4) regulates homologous recombination DNA repair, and its deficiency sensitizes cells to poly(ADP-ribose) polymerase (PARP) inhibitor treatment. J Biol Chem.

[36] Lathia JD, Mack SC, Mulkearns-Hubert EE, Valentim CLL, Rich JN (2015). Cancer stem cells in glioblastoma. Genes Dev..

[37] Venere M (2014). Therapeutic targeting of constitutive PARP activation compromises stem cell phenotype and survival of glioblastoma-initiating cells. Cell Death Differ.

[38] Kitange GJ (2016). Retinoblastoma Binding Protein 4 Modulates Temozolomide Sensitivity in Glioblastoma by Regulating DNA Repair Proteins. Cell Rep.

[39] Ogiwara H, Kohno T (2012). CBP and p300 histone acetyltransferases contribute to homologous recombination by transcriptionally activating the BRCA1 and RAD51 genes. PLoS One.

[40] Pegoraro G (2009). Ageing-related chromatin defects through loss of the NURD complex. Nat Cell Biol.

[41] Sims JK, Wade PA (2011). Mi-2/NuRD complex function is required for normal S phase progression and assembly of pericentric heterochromatin. Mol Biol Cell.

[42] Watts F (2016). Repair of DNA Double-Strand Breaks in Heterochromatin. Biomolecules.

[43] Fernandez-Capetillo O, Nussenzweig A (2008). ATM breaks into heterochromatin. Mol Cell.

[44] Rivera M (2015). Acquisition of meiotic DNA repair regulators maintain genome stability in glioblastoma. Cell Death Dis..

[45] Kesari S (2011). DNA damage response and repair: insights into strategies for radiation sensitization of gliomas. Future oncology (London, England).

[46] Lee J (2006). Tumor stem cells derived from glioblastomas cultured in bFGF and EGF more closely mirror the phenotype and genotype of primary tumors than do serum-cultured cell lines. Cancer Cell.

[47] Mehta S (2011). The Central Nervous System-Restricted Transcription Factor Olig2 Opposes p53 Responses to Genotoxic Damage in Neural Progenitors and Malignant Glioma. Cancer Cell.

[48] Moffat J (2006). A lentiviral RNAi library for human and mouse genes applied to an arrayed viral high-content screen. Cell.

[49] Yang X (2011). A public genome-scale lentiviral expression library of human ORFs. Nat Methods.

