# Supplementary material for: CHD4 regulates the DNA damage response and RAD51 expression in glioblastoma
Source: Sci Rep. 2019 Mar 14;9:4444. doi: 10.1038/s41598-019-40327-w (PMC6418088; doi:10.1038/s41598-019-40327-w)

CHD4 regulates the DNA damage response and RAD51 expression in glioblastoma

Lisa D. McKenzie, John W. LeClair, Kayla N. Miller, Averey D. Strong, Hilda L. Chan, Edward L. Oates, Keith L. Ligon, Cameron W. Brennan, Milan G. Chheda

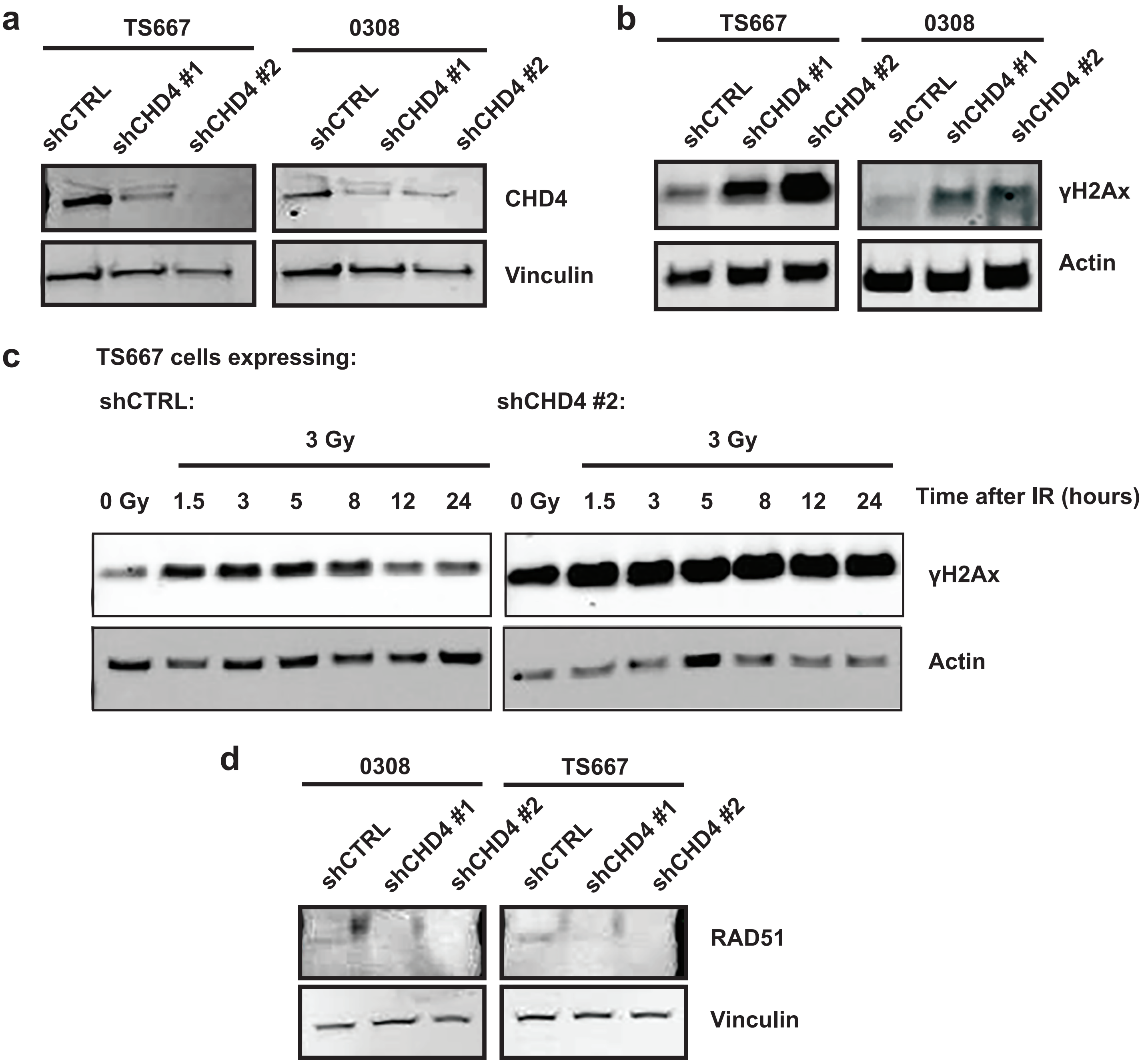

Supplementary Figure S1: CHD4 suppression in tumor initiating cells results in a defective DNA damage response and decreased RAD51 expression. (a, b) Immunoblot for CHD4 (a) and γH2Ax (b) after suppression with the indicated shRNAs in TS667 and 0308 cells. **(c)** Immunoblot for γH2Ax in TS667 cells expressing shCTRL or shCHD4#2 after either no radiation (0Gy) or 3Gy radiation at 1.5, 3, 5, 8, 12 or 24 hours after exposure. **(d)** Immunoblot for RAD51 after suppression of CHD4 using the indicated shRNAs.

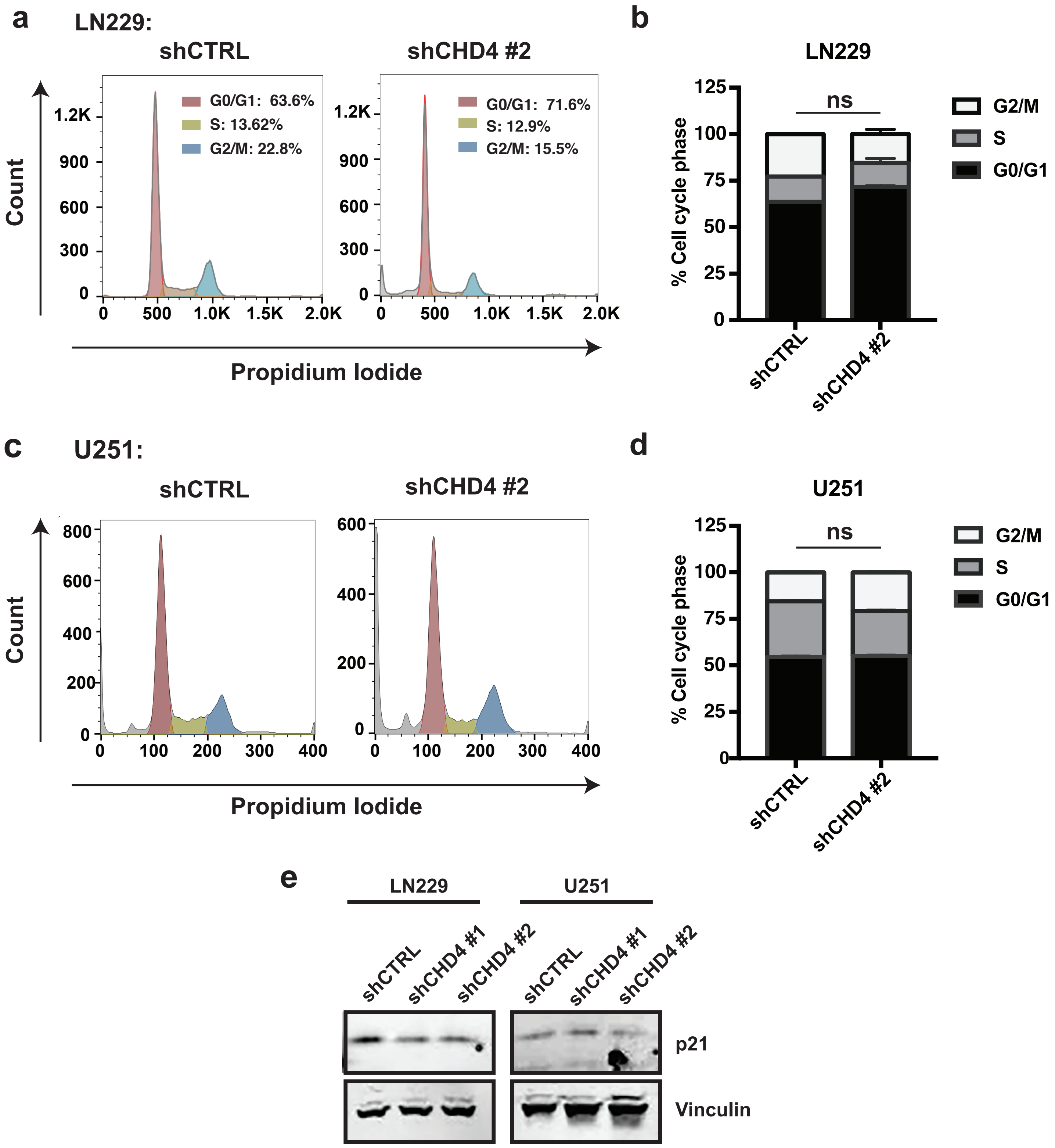

Supplementary Figure S2: Loss of CHD4 in LN229 and U251 cells does not significantly alter cell cycle. (a, b) Cell cycle profile of LN229 cells expressing shCTRL or shCHD4 #2 (a), which is graphed in (b). **(c, d)** Cell cycle profile of U251 cells expressing shCTRL or shCHD4 #2 (c), which is graphed in (d). Average percent of cells in each phase is indicated. Data are shown as the mean ± SEM. **(e)** Immunoblot for p21 protein expression after depletion of CHD4 in the indicated cell lines.

Supplementary Figure 3:
Full scans of western blots

Fig 1c

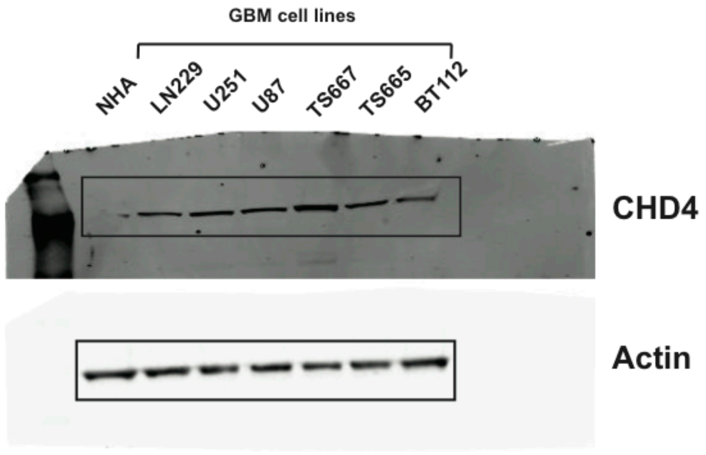

Fig 2a

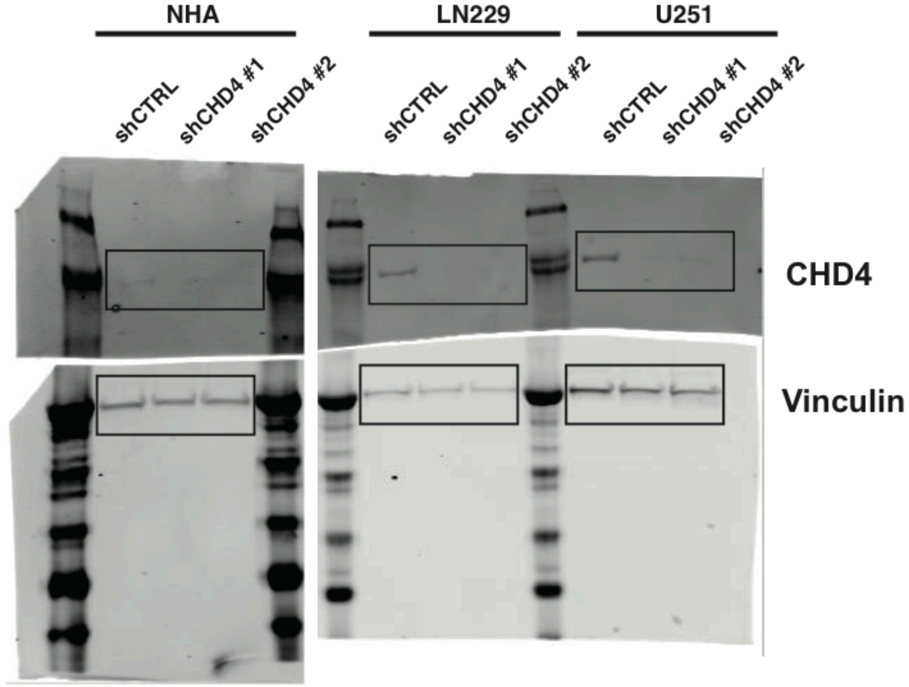

Fig 3a

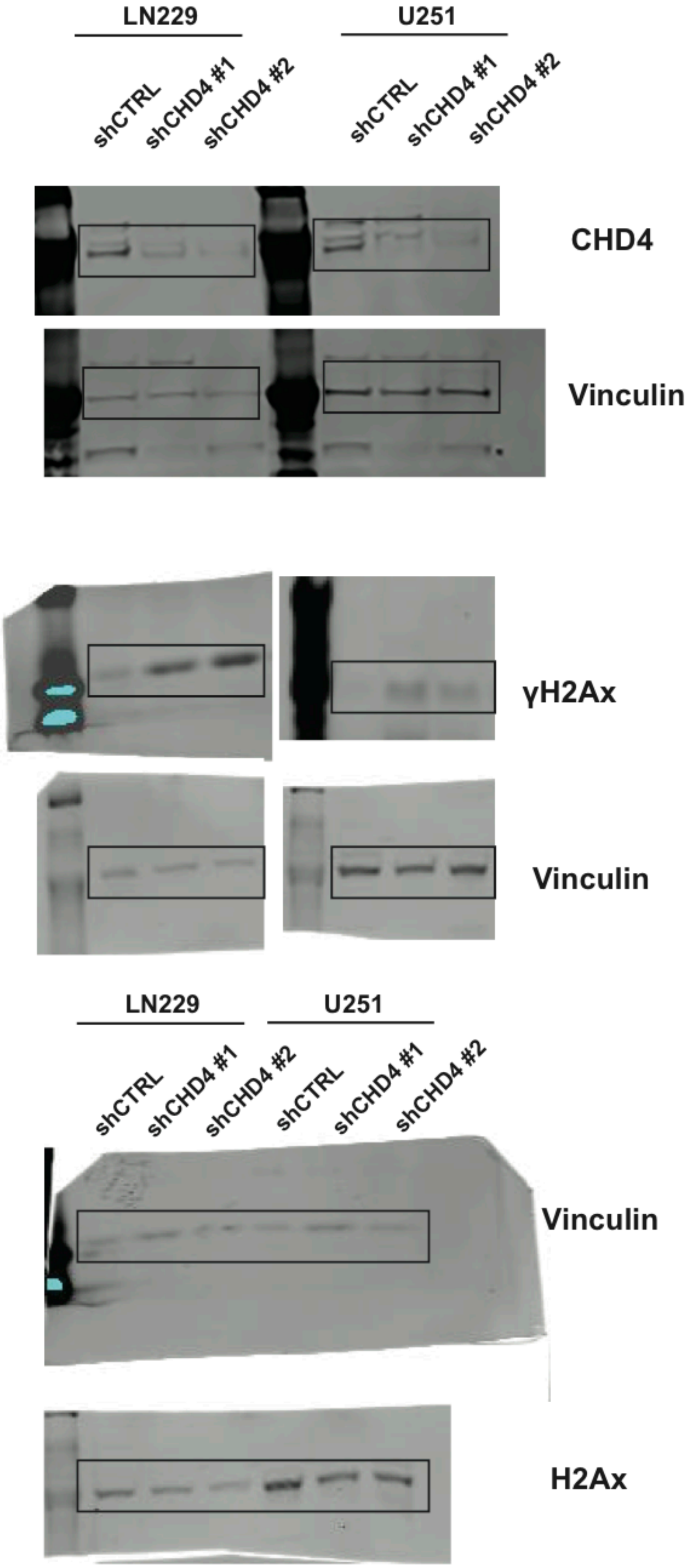
LN229
U251
shCTRL
shCHD4 #1
shCHD4 #2
shCTRL
shCHD4 #1
shCHD4 #2
CHD4
Vinculin
γH2Ax
Vinculin
LN229
U251
shCTRL
shCHD4 #1
shCHD4 #2
shCTRL
shCHD4 #1
shCHD4 #2
Vinculin
H2Ax

Fig 3c

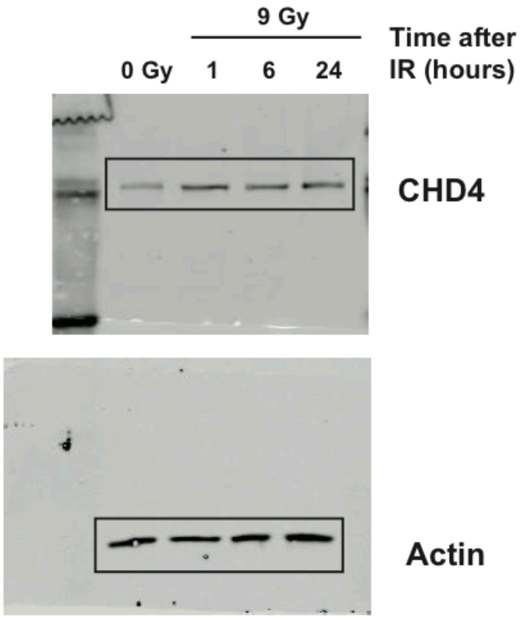
9 Gy
Time after IR (hours)
0 Gy
1
6
24
CHD4
Actin

Fig 3e

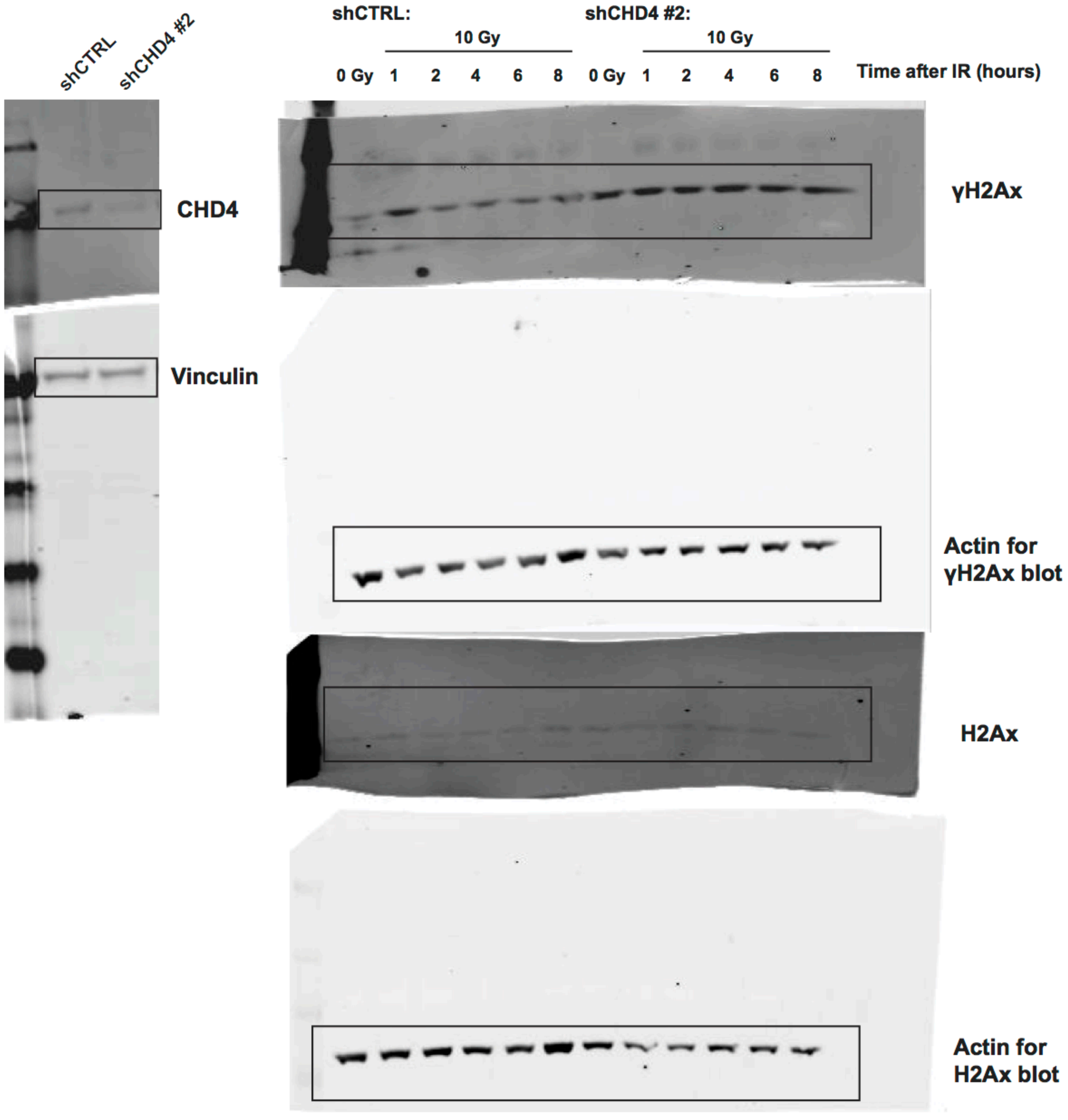
shCTRL
shCHD4 #2
CHD4
Vinculin
shCTRL:
shCHD4 #2:
10 Gy
10 Gy
0 Gy 1 2 4 6 8 0 Gy 1 2 4 6 8 Time after IR (hours)
γH2Ax
Actin for γH2Ax blot
H2Ax
Actin for H2Ax blot

Fig 4a

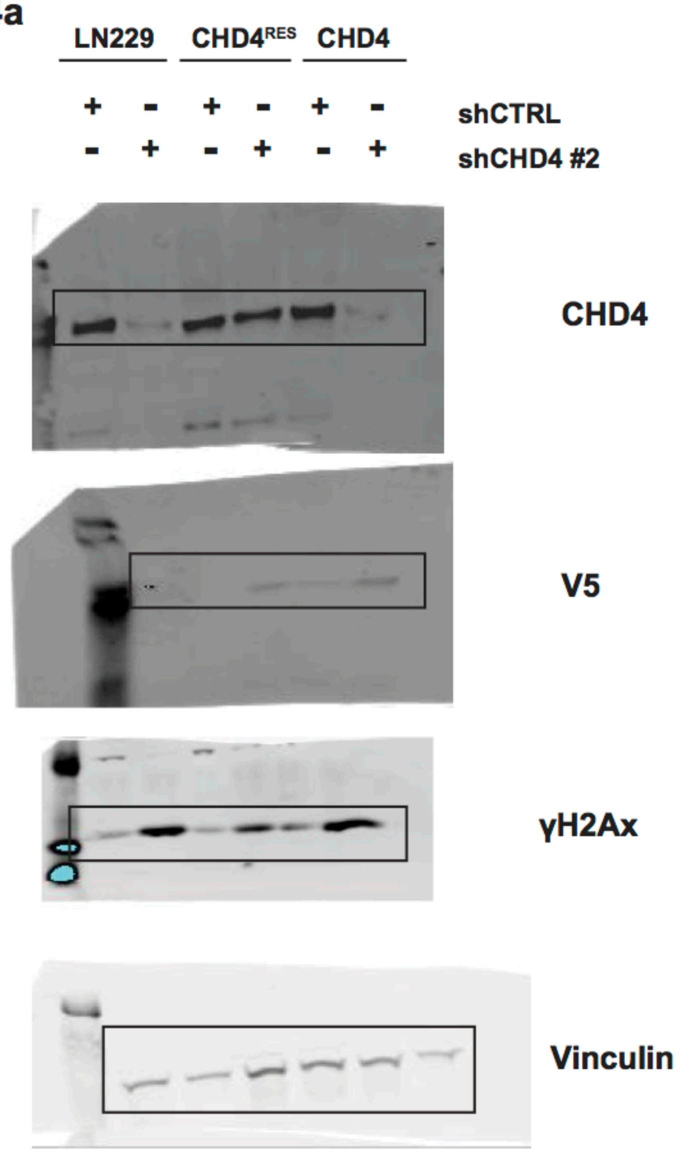

Fig 5b

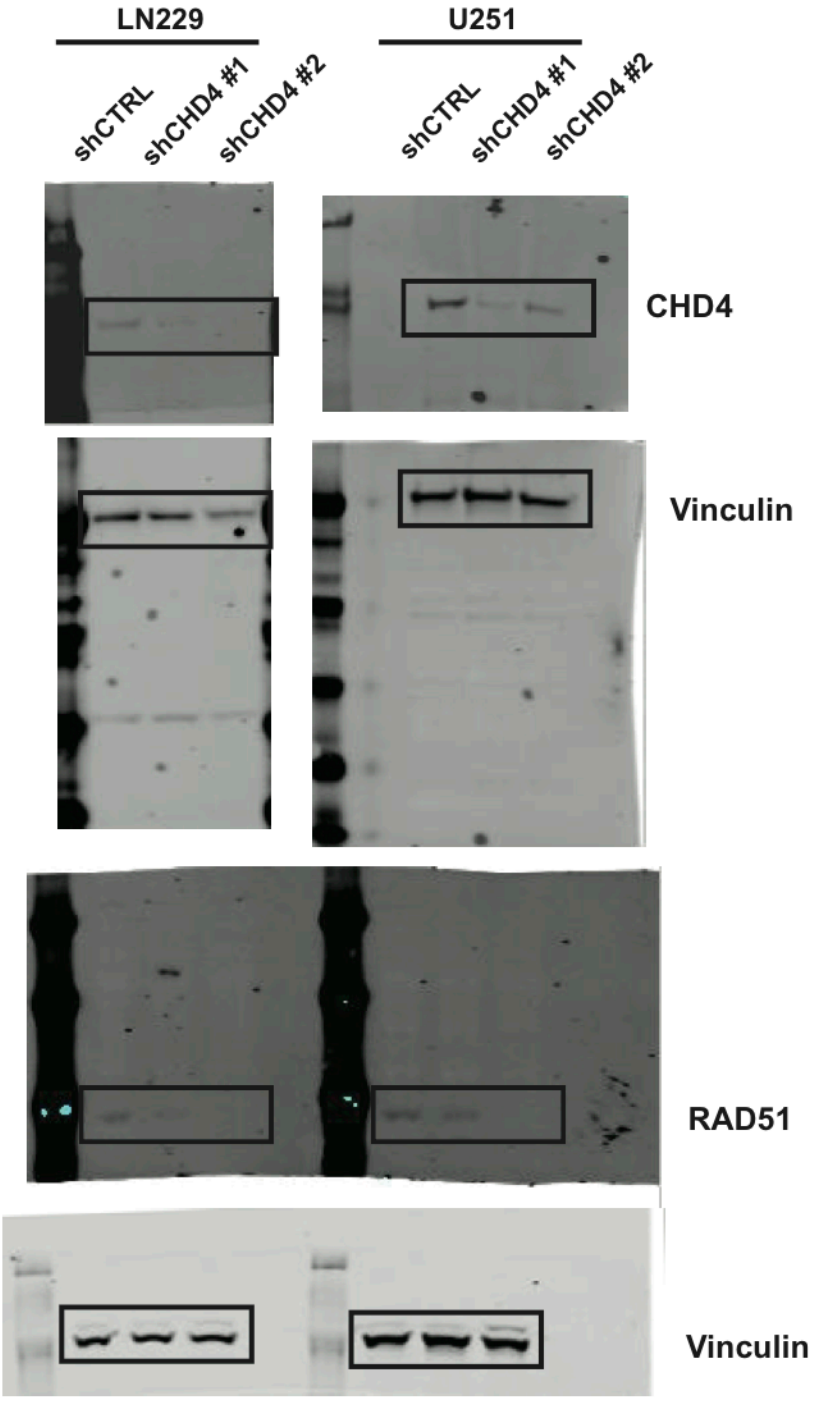

LN229
U251
shCTRL
shCHD4 #1
shCHD4 #2
shCTRL
shCHD4 #1
shCHD4 #2
CHD4
Vinculin
RAD51
Vinculin

Supplemental figure 1a

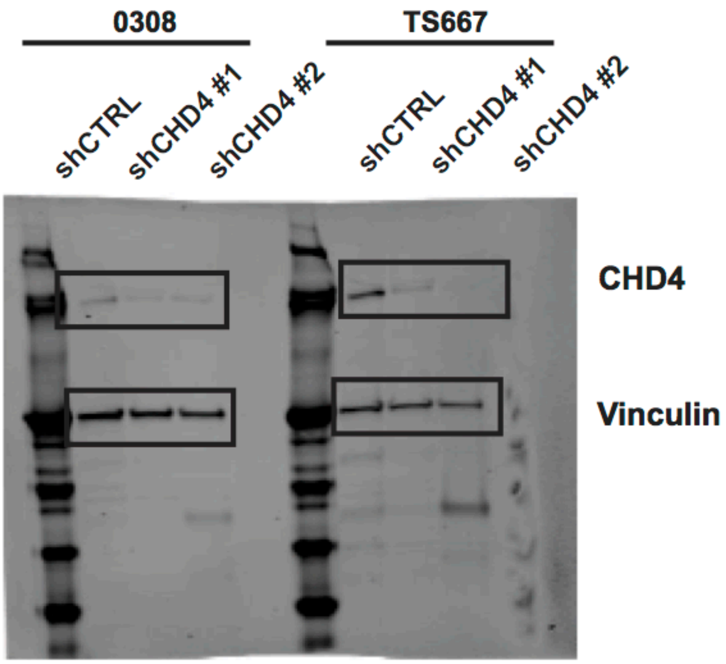

Supplementary Figure 1b

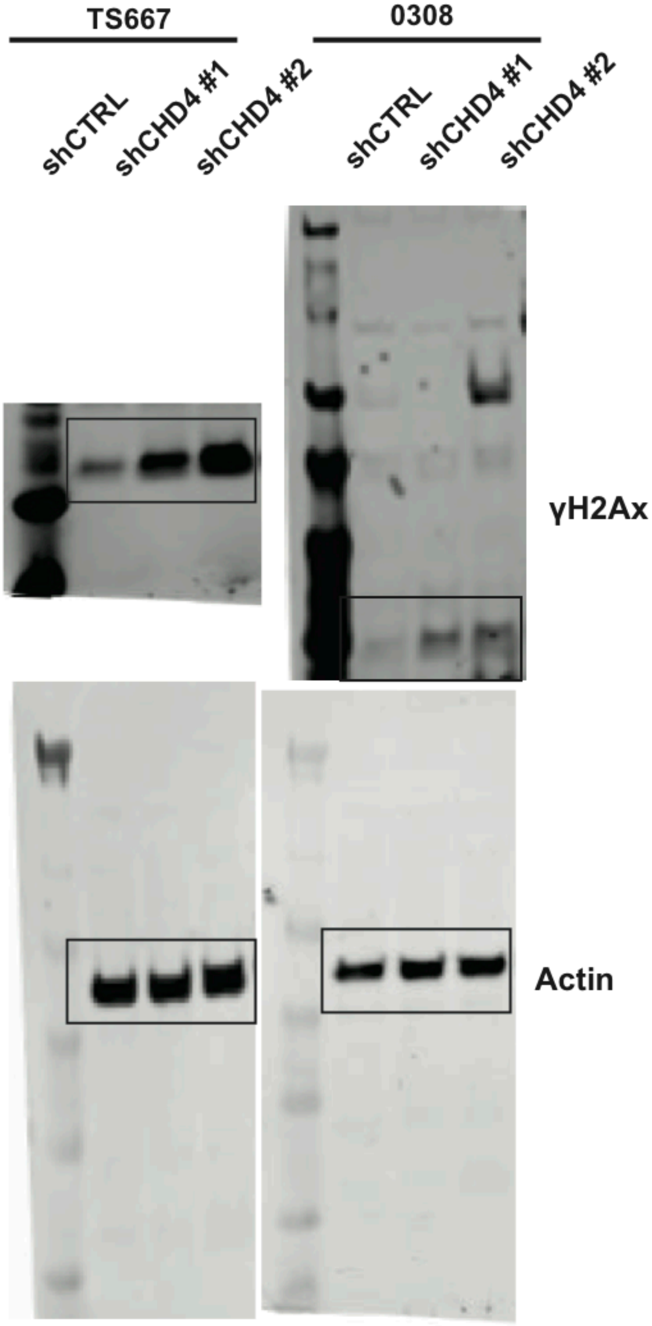

Supplementary Figure 1c

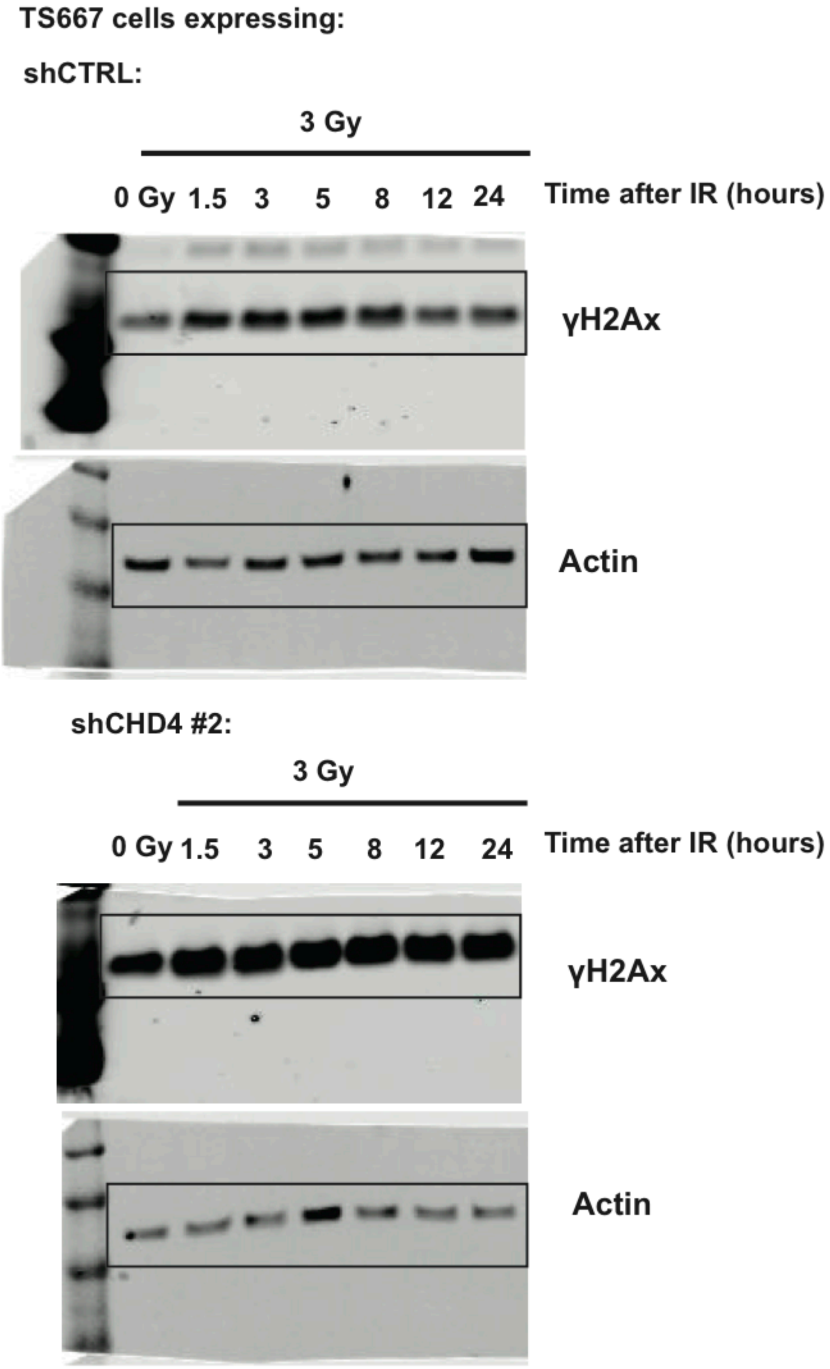

Supplementary Figure 2e

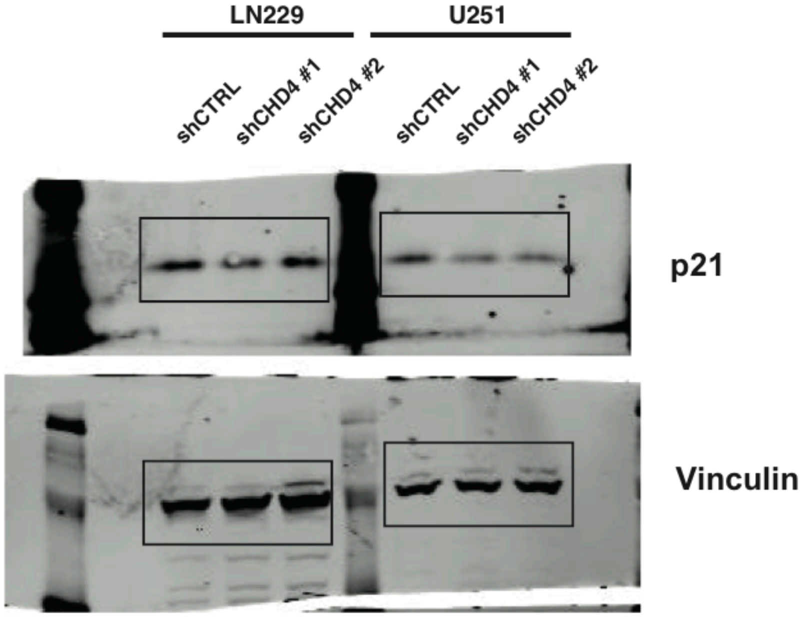

Supplement: Supplementary file 1 — Supplementary figures [file 41598_2019_40327_MOESM1_ESM.pdf]
